# Parsing the Flanker task to reveal behavioral and oscillatory correlates of unattended conflict interference

**DOI:** 10.1038/s41598-019-50464-x

**Published:** 2019-09-25

**Authors:** Marcella Brunetti, Filippo Zappasodi, Pierpaolo Croce, Rosalia Di Matteo

**Affiliations:** 1 Department of Neuroscience, Imaging and Clinical Sciences, University “G d’Annunzio”, Chieti, Italy; 2 Institute for Advanced Biomedical Technologies, Chieti, Italy

**Keywords:** Attention, Cognitive control

## Abstract

Stimulus-Response conflict is generated by an overlap between stimulus and response dimensions, but the intrinsic nature of this interaction is not yet deeply clarified. In this study, using a modified Eriksen flanker task, we have investigated *how* flankers have to be incongruent to target in order to produce an interference and *whether* and *how* this interference interacts with the one produced by Stimulus features overlap. To these aims, an Eriksen-like task employing oriented hands\arrows has been designed to distinguish between two types of Stimulus-Response (S-R) interferences: one derived by a short-term association and one based on automatic processes. Stimulus-Stimulus (S-S) conflict has been also included in the same factorial design. Behavioral, Event Related Potential (ERP) and oscillatory activity data have been measured. Results revealed distinct S-S and automatic S-R effects on behavioral performance. ERP and Theta band power modulation results suggested an early frontal S-S conflict processing followed by a posterior simultaneous S-S and automatic S-R conflict processing. These findings provide evidence that, in presence of different conflicts, the sequence of stimulus identification and response selection could not move forward in a linear serial direction, but it may involve further effort, mirrored in posterior late components and response time prolongation.

## Introduction

In the last three decades, several studies have been conducted into the frame of cognitive control to investigate the dynamic of conflict monitoring. Following the Kornblum Dimensional Overlap (DO) model, matches and mismatches between the irrelevant stimulus and the relevant stimulus or response are called Stimulus-Stimulus (SS)- or Stimulus-Response (SR)-congruency and incongruency, respectively^1–3^. The incongruence generates a cognitive conflict. The S-S conflict is supposed to have an impact on the stimulus-encoding stage, whereas the S-R conflict might affect the response-selection stage. A still debated question is whether a general domain or specific mechanisms underlie the two conflicts, i.e. whether the two processes have a serial or a parallel nature. Few studies have combined different conflict types in a fully factorial design, thus making hard to test whether conflict-driven control is domain-general or domain-specific^4^.

Furthermore, the S-R conflict is, by definition, generated by the interaction between stimulus and response, but the intrinsic nature of this interaction has not yet been clarified. An exhaustive investigation of this problem should consider the nature of the stimulus used, that could automatically activate a response or, conversely, require voluntary recall of short-term memory information.

In this frame, several standard and modified versions of well-known tasks (e.g. Stroop and Simon tasks) have been used to investigate Stimulus-Stimulus and Stimulus-Response interaction. Among the different Stimulus-Response Conflict (SRC) tasks traditionally employed, the Eriksen flanker task is widely used^5,6^. In the classic version of the Eriksen flanker task, a string of letters is shown to participants, who are instructed to press a key according to the central letter (target letter): two different letters are associated to a same key, and other two letters to another key. Crucially, the target letter could be alternatively flanked by letters belonging to the same key (congruent condition) or to the other key (incongruent condition). Typically, reaction time (RT) is faster when the central and the flanking letters are mapped to the same answer key rather than when the answer keys are different. This 2-1 mapping (two stimuli mapped to the same response) allowed to manipulate both S-S conflict (target and flankers are different but assigned to the same response) and S-R conflict (target and flankers are different and assigned to different responses). Consequently, the former conflict in this task should elicit interference between stimulus features of target and flanker stimuli (S-S), whereas the latter should lead to response competition (S-R)^7^. Importantly, in this version of the task, stimuli and response are associated only as the result of task instruction, namely by means of a short-term memory association^8^.

A subsequent version of the Eriksen task has been employed by manipulating left or right oriented arrows^9^. In this variant, participants are typically presented with a five arrows array having same orientation (<<<<<) or different orientation between target and flankers (<<><<). Participants are asked to answer depending on the orientation of target (central arrow), thus facing a S-S conflict when target and flanker are differently oriented. Actually, arrows are supposed to be processed automatically and involuntarily since they are overlearned symbols for direction^9,10^. As suggested by the dimensional overlap model^1,7^, arrows automatically activate a response due to a dimensional overlap between stimulus and response (spatial dimension). This variant then allowed both to manipulate the visuo-spatial component of stimulus processing beyond conflict monitoring mechanisms^11^ and to investigate automatic process underling the S-S conflict. Nevertheless, the distinction among conflicts based on S-S, short-term S-R and automatic S-R overlaps, as well as their putative mutual interactions, are difficult to study, since it requires a paradigm with a factorial design that should include all the three conflicts. Indeed, an ad-hoc manipulation is required to verify the independent or interactive nature of these processes.

Going in this direction, previous studies investigated the effect of interference in Eriksen combined with stimulus-response tasks, as the Simon task. Mansfield and colleagues^12^ collected behavioral data and Event Related Potentials (ERPs) to compare interference in Eriksen and in Simon tasks and concluded that these two types of interference effects are based on different processes, as described by different ERP patterns between the two tasks^12^. Frühholz and colleagues^13^ combined Eriksen and Simon tasks in a functional Magnetic Resonance Imaging (fMRI) and ElectroencEphaloGraphic (EEG) study, demonstrating distinct mechanisms underlying S-S and S-R conflict. During double conflict condition, S-S conflict was mainly processed by anterior frontal regions and modulated the N2 component of ERP, whereas S-R conflict elicited a parietal P3b component. Given the absence of time overlap of the two components, authors suggested a sequential processing. A similar double conflict was also studied by Peschke and colleagues^14^ by means of fMRI and repetitive Transcranial Magnetic Stimulation (rTMS). The authors demonstrated that the two different conflicts activate distinct brain regions and are selectively perturbed by TMS^14^. However, the hypothesis of independence and then of the serial nature of these processes does not meet a total consent, since doubts on the independence of these processes have been raised^15^ (see^4^ for a review).

Even if the cited studies have successfully combined S-S and S-R conflicts, the simultaneous combination of conflicts based on both short- and long-term information competitions remains challenging. In other words, tasks commonly employed to investigate the SRC effect are based on long-term association between correct response and stimulus features, or alternatively, on short-term association between them. A combination of these features in a fully factorial design could be helpful to deeply investigate the S-R conflict effect and its possible interaction with the S-S conflict, but, as far as we know, it has still not been tempted elsewhere.

Electrophysiological measures aiming to compare interference in the Eriksen and Simon tasks might clarify the extent to which these similar forms of interference affect the same processes. Specifically, the most frequently observed electrophysiological correlates of cognitive control are the N2 and P3 components. N2, a negative ERP component peaking at about 200 ms following stimulus onset, is assumed to reflect the selection of the appropriate response^16^ and conflict processing^17–20^. When conflict in incongruent trials is resolved, leading to a correct response, N2 amplitude is often enhanced with respect to correct congruent trials^9,12,19^. Furthermore, several researchers demonstrated that, during congruent S-R condition, the amplitude of the parietal P3, was significantly greater than those during incongruent condition^21–24^.

Finally, growing evidence on cognitive control research suggested that frontal theta oscillatory activity (4–7 Hz) reflects a generic device of action-monitoring, by communicating with other crucial brain structures for behavioral adjustment^25,26^.

In the present study we attempted to specify *how* flankers have to be incongruent to target in order to produce an interference and *whether* and *how* this interference interacts with the interference deriving from Stimulus features overlap. Specifically, we aimed at investigating if the short-term response code associated to flankers interferes with automatically generated responses (i.e. those driven by flanker direction) or with short-term learned responses (i.e. response code associated to target). Our purpose was also to describe the temporal dynamics of the mechanism elicited by these processes. To these aims, a novel version of the Eriksen Flanker task has been designed and ERPs and modulation of brain oscillatory activity by means of EEG have been measured.

**Figure 1:**
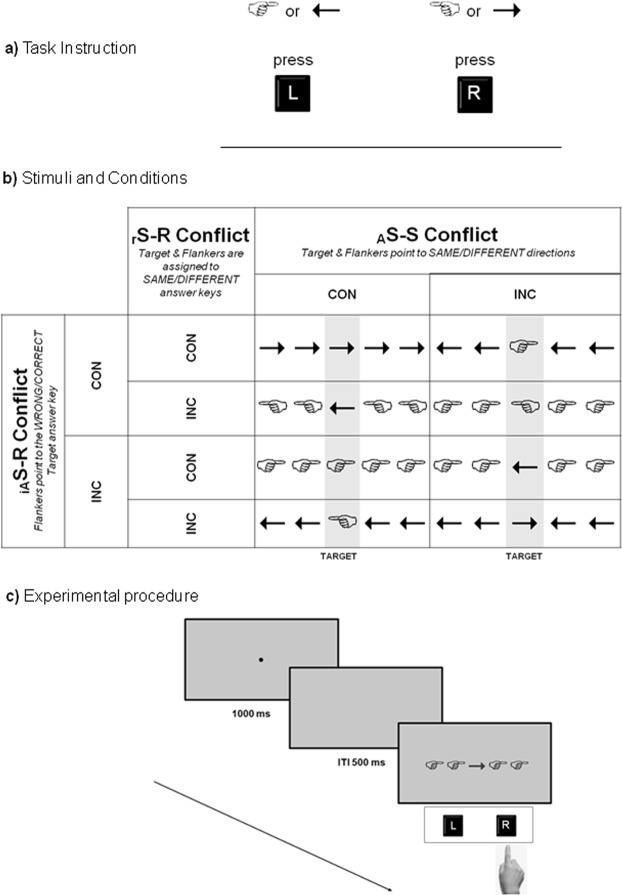
(**a**) Instructions to participants: press the key “R” when either left hand or right arrow were presented as target, and the key “L” when either right hand or left arrow were presented as target. Previous instructions could be delivered randomly either in the first or in the second half of the experiment; reversed instructions, with inverted answer keys, were delivered in the other half of the experiment. Block order was counterbalanced across participants (**b**) Example of experimental stimulus and conditions according to the instructions described in a). The same mirrored schema has been applied to the inverted answer keys. The three manipulated conditions were: relevant Stimulus - Response S-R (_*r*_*S-R*) conflict, Automatic Stimulus-Stimulus (_A_*S-S*) conflict and irrelevant Automatic Stimulus - Response (_*iA*_*S-R*) conflict; CONG: congruent, INC: incongruent. (**c**) Illustration of the experimental procedure.

The investigation on whether same or different origins are at the basis of different conflicts could suffer from different nature of the stimuli (i.e. color vs meaning for the Stroop and color vs spatial position for the Simon). Consequently, in our study we reduced the distance between stimuli and included them in a full-factorial design: in our paradigm, the three conflicts were all based on arrows or hands with differently combined orientations. Specifically, the relevant short-term conflict (_r_S-R) derived by task instruction (answer key associated to left-right oriented arrow/hand as target vs answer key associated to left-right oriented arrow/hand as flankers); the automatic Stimulus-Stimulus conflict (_A_S-S) was based on congruency between target and flankers left-right orientation; finally, irrelevant automatic Stimulus-Response conflict (_iA_S-R) derived by congruency between flankers left-right orientation and left-right location of the answer key (Fig. 1).

## Results

### Behavioral results

After recordings, behavioral data from one out the 21 subjects were unreadable due to technical problems. Consequently, the final behavioral analysis was performed on a total of twenty subjects. As a first control analysis, to exclude confounding effect of the different side of the answer keys between the 2 blocks, a first Repeated Measures Analysis of Variance (ANOVA) was performed to compare RTs and accuracies of the two blocks. Since no differences were evidenced (p > 0.05), the 2 blocks were collapsed and the RTs and correct answers of the whole experiment were considered for further analysis.

Repeated measures ANOVAs were applied to RT and accuracy. A 2 × 2 × 2 design was carried out, with relevant Stimulus-Response (_r_S-R) (congruent and incongruent: hereafter C and I), Automatic Stimulus- Stimulus _A_S-S (C and I) and irrelevant Stimulus-Response _iA_S-R (C and I) as within subject factors.

#### RT

**Figure 2:**
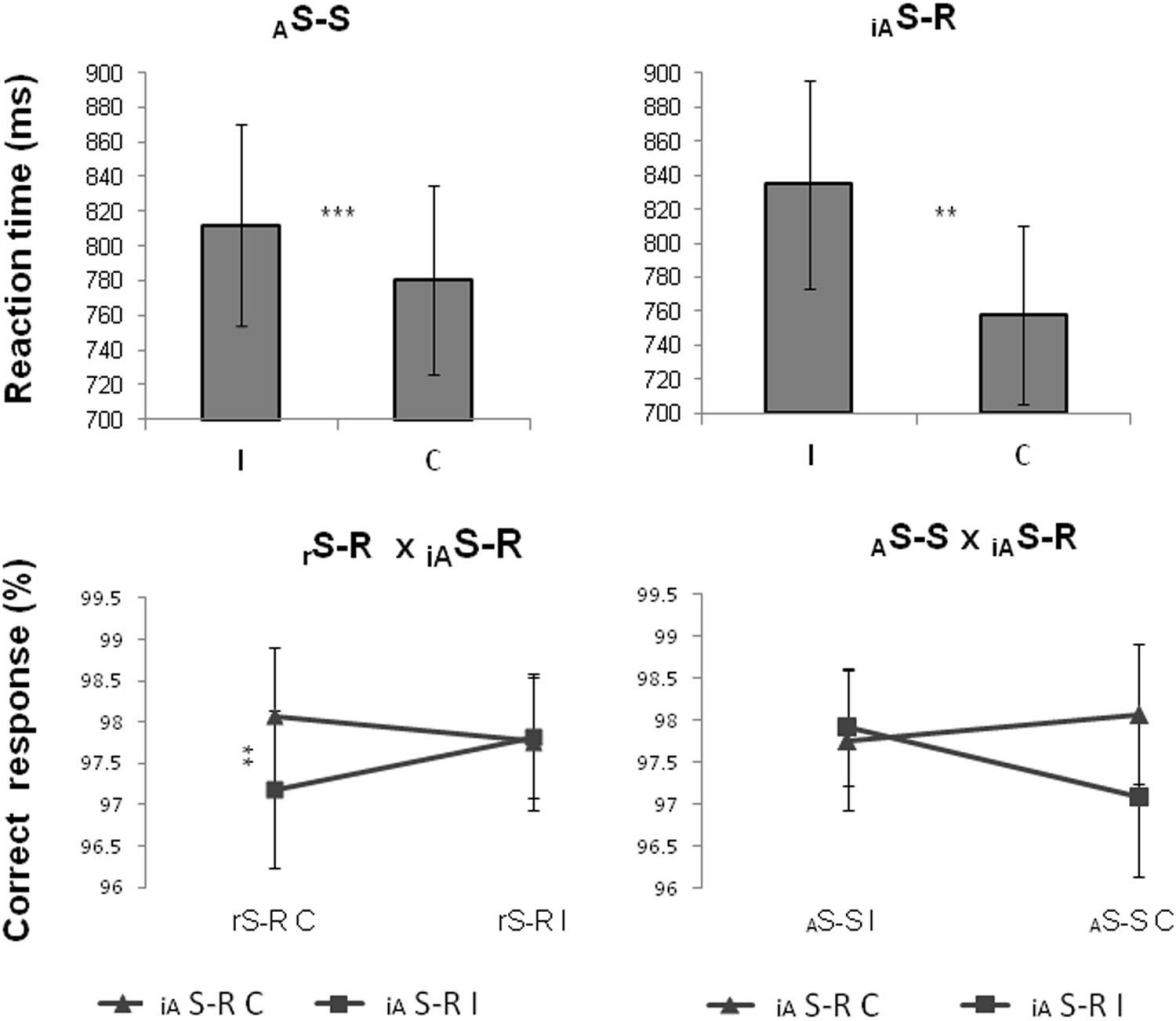
*Top*: Mean and standard errors of RT in _A_S-S (C and I) and _iA_S-R (C and I). *Bottom*: Mean and standard errors of accuracy for _r_S-R, _A_S-S and _iA_S-R. Post Hoc Bonferroni correction p < 0.05. _A_*S-S:* Automatic Stimulus-Stimulus conflict; _*iA*_*S-R:* irrelevant Automatic Stimulus – Response conflict; _*r*_*S-R:* relevant Stimulus - Response conflict; C: congruent, I: incongruent. **p < 0.001; ***p < 0.0005.

ANOVAs on the corrected RTs revealed a main effect of _A_S-S [*F* (1, 19) = 20.67, *p* < 0.0005; η_p_^2^ = 0.521; C.I. = 0.224–0.671; observed power = 0.99] with a performance significantly slower during incongruent than congruent trials. Furthermore, a main effect of _iA_S-R [*F* (1, 19) = 15.46, *p* < 0.001; η_p_^2^ = 0.449; C.I. = 0.152–0.619; observed power = 0.96] with a performance significantly slower during incongruent than congruent trials was observed (Fig. 2, Top). No interactions were observed.

#### Accuracy

On the accuracy, no main effects were observed. Two interaction effects between two factors were observed. First, a significant interaction between _r_S-R and _iA_S-R was found [F (1, 19) = 5.94, p < 0.05; ηp^2^ = 0.238; C.I. = 0.018–0.454; observed power = 0.63]. Bonferroni post hoc revealed that during the congruent _r_S-R trials, incongruent _iA_S-R performance was worse than the congruent one (p < 0.05). No differences were observed for the incongruent _r_S-R condition (Fig. 2, Bottom). Second, a significant _A_S-S x _iA_S-R interaction [F (1, 19) = 5.76, p < 0.05; ηp^2^ = 0.233; C.I. = 0.016–0.449; observed power = 0.62] was observed, explained by a trend (p = 0.06) to a better performance when both _A_S-S and _iA_S-R were congruent than during the other conditions (Fig. 2, Bottom).

### EEG results

**Figure 3:**
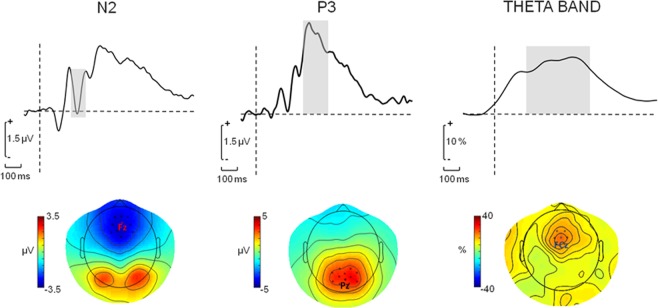
Time course of the average of frontal (close to Fz), parietal (close to Pz) and fronto-central (close to FCz) channels used to obtain amplitude of N2, P3 and theta band modulation. The time windows where the ERP components and band power values were computed are shown in grey. Horizontal line corresponds to the time of stimulus presentation. The map topographies of EEG potential values and theta band power changes in the evidenced time windows are shown.

Repeated measures Analyses of Variance (ANOVAs) were applied to both latencies and amplitudes of N2, estimated over the frontocentral EEG channels around FCz, and of P3, estimated over centroparietal EEG channels around PZ (Fig. 3). A 2 × 2 × 2 design was carried out, with _r_S-R (C and I), _A_S-S (C and I) and _iA_S-R (C and I) as within subject factors. The same statistical design was applied to frontocentral theta band power modulation (Fig. 3).

#### ERP latency

The observed mean N2 and P3 peak latencies across all subjects and conditions were 256 ± 53 ms and 360 ± 46 ms respectively. The mean latency of the two components was statistically different (paired t-test p < 0.00001 for all conditions). For both N2 and P3 latencies ANOVAs revealed no statistical significant effects (consistently p > 0.2).

#### ERP amplitude

**Figure 4:**
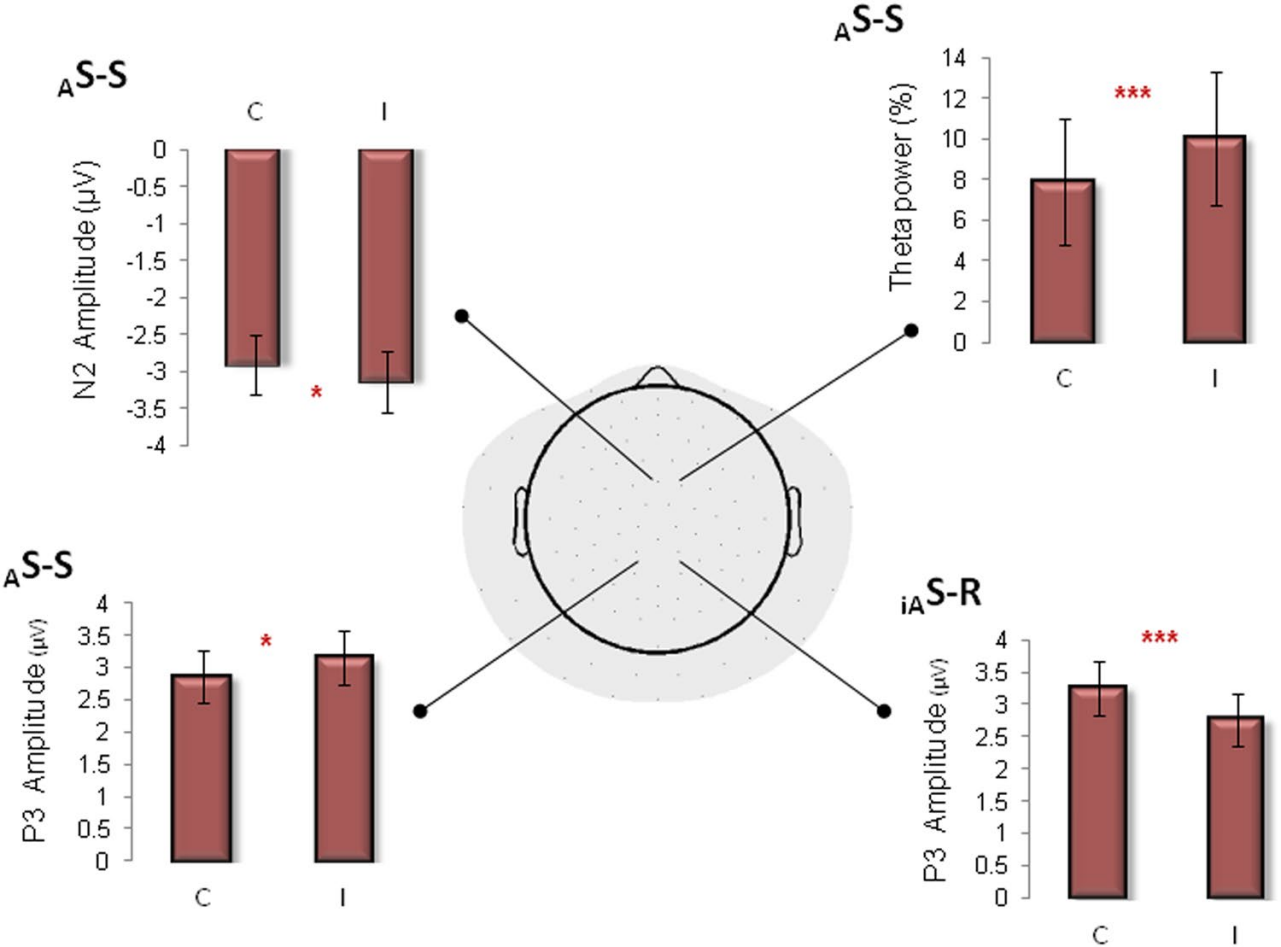
Bar plots show average amplitude and TFR values across conditions. Main _A_S-S effect was observed for N2 (top left) and theta band power (top right) over frontocentral FCz electrode. For P3 amplitude over Pz, main effects of both _A_S-S (bottom left) and _iA_S-R conflicts (bottom left) are shown. Post Hoc Bonferroni correction p < 0.05. _A_*S-S:* Automatic Stimulus-Stimulus conflict; _*iA*_*S-R:* irrelevant Automatic Stimulus – Response conflict; C: congruent, I: incongruent. *p < 0.05; ***p < 0.0005.

For N2 component, the main effect of _A_S-S was significant, with more negative N2 amplitude to incongruent than to congruent trials [*F* (1, 20) = 4.64, *p* < 0.05; η_p_^2^ = 0.189; C.I. = 0.003–0.405; observed power = 0.54]. N2 amplitude results are displayed in Fig. 4.

Also for the P3 amplitude, ANOVA analysis revealed a _A_S-S main effect with P3 more positive during incongruent that congruent trial [*F* (1, 20) = 5.01, *p* < 0.05; η_p_^2^ = 0.2; C.I. = 0.007–0.416; observed power = 0.57]. Furthermore, a main effect of _iA_S-R was observed, with a reverse pattern: P3 amplitude was grater during congruent than incongruent _iA_S-R trials [*F* (1, 20) = 17.84, *p* < 0.0005; η_p_^2^ = 0.47; C.I. = 0.181–0.632; observed power = 0.98] as shown in Fig. 4. For both periods, no significant interaction was observed.

#### Theta band results

The result of the _A_S-S effect on theta band synchronization is displayed in Fig. 4. ANOVA analysis revealed a _A_S-S main effect: Theta band power was enhanced for incongruent than congruent _A_S-S trials [*F* (1, 20) = 4.95, *p* < 0.05; η_p_^2^ = 0.198; C.I. = 0.198–0.414; observed power = 0.56]. Neither other main effects nor interactions were observed.

All results are summarized in Table 1

**Table 1:** Mean behavioral and EEG results for all conditions.

	rS-S Conflict							
	*Congruent*				*Incongruent*			
	S-S Conflict							
	*Incongruent*		*Congruent*		*Incongruent*		*Congruent*	
	_iA_S-R Conflict							
	*Inc*	*Con*	*Inc*	*Con*	*Inc*	*Con*	*Inc*	*Con*
RT(ms) Mean (SE)	844 (58)	762 (57)	813 (65)	747 (51)	861 (67)	781 (58)	820 (60)	742 (46)
Accuracy (%) Mean (SE)	97.3 (0.5)	97.9 (0.4)	97.1 (0.5)	98.2 (0.4)	98.5 (0.2)	97.6 (0.4)	97.1 (0.5)	97.9 (0.4)
N2 ampl (μV) Mean (SE)	−2.9 (0.5)	−3.1 (0.6)	−2.8 (0.6)	−2.9 (0.6)	−3.5 (0.6)	−2.9 (0.6)	−3.0 (0.5)	−2.8 (0.6)
P3 ampl (μV) Mean (SE)	3.1 (0.4)	3.4 (0.5)	2.4 (0.4)	3.1 (0.4)	2.8 (0.4)	3.2 (0.4)	2.7 (0.4)	3.3 (0.5)
Theta power (%) Mean (SE)	12.2 (0.4)	10.8 (0.4)	8.2 (0.4)	6.2 (0.4)	8.3 (0.4)	8.9 (0.4)	7.7 (0.4)	9.3 (0.4)

Note: RT: Reaction time; SE: Standard Error; Inc: Incongruent; Con: Congruent.

## Discussion

In the present study we conducted behavioral and EEG experiments in order to investigate the effect of both Stimulus-Stimulus and Stimulus-Response conflicts on target response. Specifically, we aimed at distinguishing between the impact exerted by a short-term S-R association interference and one based on automatic processes on target response. Furthermore, the manipulation that we made allowed us to include in the same factorial design these two kinds of S-R conflicts and a S-S conflict condition, in order to investigate their potential mutual interaction.

Altogether, our results revealed distinct automatic S-S and automatic S-R effects on behavioral performance; furthermore, a complex EEG pattern was observed, suggesting an early frontal S-S conflict processing followed by a posterior simultaneous S-S and automatic S-R conflict processing.

Our behavioral results indicate two distinct main effects: on one hand, automatic Stimulus-Stimulus conflict (namely, _A_S-S incongruent condition) slowed down performance, on the other hand a main effect of irrelevant automatic S-R conflict was observed. No significant interactions were present in reaction time analysis. The first result could be considered in agreement with previous findings using Flanker or Stroop tasks. Indeed, several studies consistently observed increased response time during S-S incongruent condition than during the congruent one^5,8,12,14,27^. Regarding the automatic S-R effect, it is crucial to remind that this kind of interference was based on the incongruence between flanker direction and response associated to flanker, and, since flanker was a space oriented symbol, the interference was based on a spatial incongruence between flanker direction and answer key side. Differently from our relevant short-term association condition (i.e. _r_S-R), where the consistency between response associated to target and response associated to flanker was manipulated, here an irrelevant stimulus features has been varied, namely flanker direction. Actually, it might be harder to ignore a distracting arrow than a target arrow and the direction information it conveys, and, thus, when this is opposed to response side, conflict might be increased. Interestingly, this condition represents a task-unrelated conflict that, on the basis of performance results, affects the goal-directed behavior. Furthermore, this feature (flanker direction) was a “to be ignored” information. Even if a direct test of conscious perception of flankers was not made, we could assume that the representation of this conflict was somehow degraded. This interpretation was in line with recent findings from Padrão and co-workers^28^. Using a modified Flanker task, the authors investigated the behavioral and EEG correlates of attended and unattended stimuli. In their task, both target and flankers were placed in parafoveal position. Behavioral results clearly indicate that, over the attended one, unattended conflict was also able to impair performance. Even if in our study the absence of main effects on accuracy undermines the observed interactions importance, the interaction between the two S-R conditions could support the hypothesis that automatic S-R interference was able to impair performance.

Finally, a preponderant effect of relevant Stimulus-Response conflict was not observed, thus disentangling the issue raised in the introduction: short-term response code associated to flankers interfered with automatically processed information (i.e. flanker direction) and not with short-term learned responses (i.e. response code associated to target). It could be that the complex design applied in our study, that includes three different types of conflict, was not sensitive enough to detect rS-R conflict. Actually, the effect of this conflict (rS-R) has been commonly observed by using non-directional stimuli (i.e. letters, see^5^). It could be argued that the short-term memory learned response associated to flankers impact more weakly on the performance with respect to the automatically generated response driven by flankers direction. This result was in line with previous findings that indicate that arrows, being overlearned symbols of direction, are processed with less cost and are therefore more easily associated to a direction and a required response than other stimuli^10^. An alternative explanation of this finding could be searched in the frame of the Lavie’s Load Theory of Attention. According to this view, under high perceptual load, the interference effect from distractors is reduced^29,30^. Actually, our complex manipulation of differently oriented hands and arrows was perceptually demanding. Following Lavie’s model, the perceptual selection mechanism that allows for excluding irrelevant distractors from perception under high perceptual load is a passive mechanism, whereby irrelevant distractor interference is prevented simply because the distractors are not perceived when there is insufficient capacity for their processing. Our rS-R conflict, the only conflict of the three presented that was based on a short-term memory association, could have engaged full cognitive capacity, thus leaving no residual capacity for distractors processing. In this line, the interaction observed between _iA_S-R and rS-R accuracy, even if not supported by main effects, further encourages this interpretation, since indicates that rS-R conflict improves performance of _iA_S-R condition. Similar results comes from previous studies using emotional conflicts and Flanker task^31^ and emotional Stroop^32^. In both studies, emotional conflict reduced the distractor interference effect. Moreover, to definitively include our findings into the Lavie’s theory, future research should be employed by adequately manipulate cognitive and perceptual load into the same factorial design.

ERP analysis revealed a medial centro-frontal involvement in cognitive conflict. Indeed, N200 amplitude, a negativity commonly sourced in the frontal medial regions, probably originating from the Anterior Cingulated Cortex (ACC^13^) was increased during incongruent S-S condition than during congruent one. This frontal component has been largely described as the electrophysiological correlate of cognitive control, and its amplitude modulation has been studied by means of several SRC tasks. Specifically, previous findings showed N200 amplitude modulation using a combined Stroop and Simon task. Authors observed a N200 amplitude additive modulation when both S-S and S-R conflicts were present, thus concluding that conflict processing was based on a domain-specific mechanism, suggesting a modular organization of cognitive conflict processing^27,33^. Along this path, Xie and colleagues^34^ tried to distinguish between three inhibition types: flanker, rule and response inhibition, respectively. Specifically, Flanker task matching with a Stimulus-Stimulus conflict has been used. Rule inhibition was referred to those tasks in which successful responses were obtained by means of suppression of irrelevant rule from working memory. Interestingly, flanker inhibition elicited larger N2 at the frontal region^34^. Furthermore, incongruent condition of Flanker task improved N200 amplitude in conflict adaptation manipulation^19^ and combined with Simon task^13^. Finally, in a fMRI-rTMS study, by combining a “direction” Flanker and a Simon task, a medial frontal cortex involvement during double conflict has been observed^14^, thus suggesting the crucial role of this area in the cognitive conflict processing.

On the other hand, previous evidences showed P3b amplitude modulation during S-R conflict trials^35,36^. In our study, this component seems to be affected by both S-S and S-R conflicts. First, a larger parietal P3 amplitude was elicited by incongruent S-S trials with respect to congruent ones. This component has been related to short-term memory updating^37^. The observed modulation could reflect the stimulus representation effort induced by conflicting direction of target and flankers. The maximal amplitude of this component was observed in parietal electrodes, in line with previously observed functional imaging activation in the superior parietal cortex during S-S single conflict condition, thus suggesting the involvement of this region in the visual information flow regulation^13,38^. In this frame, Egner and colleagues^38^ collected evidences in favor of the hypothesis that superior parietal cortex plays a crucial role in a top-down selective mechanisms aimed at biasing visual information processing to enhance task-relevant stimulus processing^38^.

Interestingly, in our study the same parietal component showed to be also affected by the automatic S-R conflict, being larger during congruent than during incongruent condition. As mentioned above, parietal P3 has been linked to short-term updating, being considered as a sign of processes of memory access that are evoked by evaluation of stimuli in tasks that require response. Several evidences suggest that P3b amplitude reflects the amount of information transmitted during presentation of a stimulus, decreasing in amplitude as memory load increases (see^39^ for a review). Considering our automatic S-R conflict, i.e. a conflict between response associated to flanker and flanker direction, one could argue that during incongruent trial of this condition, the stimulus-response mapping, learned during task instruction and stored in short-term memory, represents a strong interference to the correct response. Consequently, a short-term memory inhibition could be needed during incongruent trials to successfully face the automatic S-R conflict. Such *need* of memory inhibition could be reflected by the observed P3 amplitude decreasing. This argumentation, that looks at a parietal involvement in S-R conflict, is encouraged by evidence from fMRI that suggest a generic role of this region in regulating visual information flow, which might be especially necessary during double conflict processing^40^.

Our time-frequency analysis clearly indicate that S-S conflict modulate fronto-central theta power, that was increased during incongruent S-S trials. Recently, theta band received special attention in the cognitive control field, since it has been referred to a wide range of processing employed in cognitive tasks. Specifically, theta band amplitude enhancement has been observed during increased encoding demand^41^, memory load^42^, cognitive conflict^27,28,33,43^ and in general when increased cognitive control is needed^26,44^. Our finding was also in line with several researches that have observed frontal theta band modulation during cognitive control tasks^26–28,33,43^. Furthermore, the conflict manipulation we applied allowed to distinguish between different conflict processing. Even if previous studies showed both S-S and S-R theta band modulation^27,33^, our findings indicate a specific theta involvement in the stimulus identification stage (i.e. S-S), mirroring the behavior of N200 component showing the same topography in the fronto-medial areas. Actually, Nigbur and colleagues^43^, by means of dipole source localization, individuated different theta band maps between stimulus interference and response interference, since activity in the former was located more ventrally than the response related interference. The authors suggested that theta activity could be different between cognitive control demands and could be differently modulated by the type of cognitive interference^43^. On the other hand, Padrão and colleagues^28^ demonstrated that medial prefrontal theta oscillatory activity, commonly related to conscious processes, takes place in response to unattended (therefore automatic) conflicting events, thus hypothesizing its function in suppression and regulation of potentially inappropriate automatic response^28^. Finally, in a recent review, frontal midline theta band activity has been proposed as a mechanism common to a wide range of events that share a need for increased cognitive control. Such mechanism could act by organizing midfrontal neuronal activity and by communicating the need for control to other brain structures^26^.

Summing up our results, the frontal activity observed by means of ERP and time-frequency analysis was largely elicited by the automatic Stimulus-Stimulus conflict, thus confirming that this kind of interference, based on stimulus identity representation, primarily involved midline frontal regions. On the other hand, the crucial role of medial prefrontal regions (ACC) in conflict control by means of monitoring and detecting several types of cognitive interference in the stream of information processing has been widely considered^45^. However, our data indicate that the same interference also modulated the amplitude of a more posterior (parietal) late component, thus suggesting a putative two-stage process needed to solve this kind of conflict. Parietal P3 has been supposed to reflect the increased perceptual demand resources (see^39^). Thus, we could suppose that the stimulus identification, started earlier, should be further coded before the correct response. Nevertheless, at the same time window, an opposite P3 amplitude pattern was observed in reference to the automatic S-R conflict. Notably, in our paradigm this condition, differently from the others two, represents a conflict between an irrelevant information automatically coded (i.e. flanker direction) and a response coded as the result of task instruction, namely by means of a short-term memory association. These two contrasting codes could prevent the correct response, thus requiring the suppression of one of them, that in turn could be reflected by the P3 amplitude decrease. This inhibition, requiring a cognitive cost, would lead to performance slowing during incongruent automatic Stimulus-Response condition. Previous evidence, indicating P3 role as crucial link between perceptual analysis and response initiation, encourage this interpretation^46^.

One of the main issues raised in this field is whether different conflicts were processed by same rather than different mechanisms. When more than one conflict is present, there is an increased information monitoring demand together with an increased need to select among competing responses. Here, encouraged by the absence of factors interaction (see^47^), we could still maintain that different mechanisms mediate different conflicts resolution. In presence of more than one conflict, the sequence of stimulus identification and response selection could not move forward a linear serial direction, but additional demand for attentional or perceptual resources could involve further effort, mirrored in posterior late components and response time prolongation.

## Methods

### Participants

Twenty-one (11 females, 10 males; mean age = 24.8 years and SD = 5.9 years) students from the d’Annunzio University were enrolled in this experiment. The subjects were right-handed (Edinburgh Inventory) healthy adult volunteers, who had no previous psychiatric or neurological history. Their sight was normal or corrected to normal. The experiment was conducted with the understanding and written informed consent of each participant, according to the Code of Ethics of the World Medical Association, and the standards established by the University of Chieti Institutional Review Board and Ethics Committee. The experimental protocol was approved by the Ethics Committee of “G. d’Annunzio” University of Chieti-Pescara.

### Apparatus and stimuli

The stimuli were presented by means of E-Prime 2.0 software (Psychology Software Tools, Inc.) on a 17-inch monitor with 1024 × 768 resolution.

All stimuli consisted of an array of 5 symbols resulting from a combination of hands and\or arrows pointing leftward or rightward: [EF] or [← →] thus generating a total of 16 arrays. For each array (hereafter called stimulus), the central symbol was the target of the task, whereas the others were flankers. In all stimuli, the five symbols were displayed in the horizontal plane, with the target in the center of the screen and the four flankers on each side equidistant from the target. All stimuli were dark against a white background and subtending approximately 0.96° of visual angle in length and 0.64° in width.

### Design and procedure

Participants were asked to press one key on the keyboard when the target was either a left hand or a right arrow, and a different key when the target was either a right hand or a left arrow, thus responses were determined by symbols orientation (see Fig. 1a). The two keys were the key “2” (labeled “L”, left) and the key “8” (labeled “R”, right) located on the top horizontal line of the keyboard, hence one key was on the left side and the other one was on the right side of the keyboard. The association between answer keys and oriented symbols was delivered to the participants by means of instructions and each participant performed half of the experiment with one randomly assigned association and the other half by inverting the association, thus balancing across all the experimental conditions both the keys’ positions (i.e., left and right) and the symbols types (i.e., arrows and hands). Moreover, each of the two blocks was preceded by a practice session, thus avoiding short term memory interference between blocks.

Based on the Kornblum taxonomy that define as relevant a dimension that subjects are instructed to attend to and irrelevant a dimension to be ignored^2^, and on the nature automatic of the arrow direction detection, three conditions has been created by manipulating the relationship between target and flankers: relevant Stimulus - Response S-R (_*r*_*S-R*) conflict, Automatic Stimulus-Stimulus (_A_*S-S*) conflict and irrelevant Automatic Stimulus - Response (_*iA*_*S-R*) conflict.

To manipulate _r_S-R, we have built two response congruency conditions by varying target and flankers according to the answer keys associated to them. In the _r_S-R congruent condition, target (relevant) and flankers were associated with the same answer key; in the _r_S-R incongruent condition, target and flankers were associated with two different answer keys.

Regarding to the _A_S-S conflict, it has been manipulated by varying the consistency between the target and flanker orientation. In the _A_S-S congruent condition, flankers (hands or arrows) pointed at the same direction as the target (i.e. right hand flanked either by right hands or right arrows). In the _A_S-S incongruent condition, flankers and target pointed at different directions (i.e. a right hand flanked either by left arrow or left hands).

Finally, the _iA_S-R conflict has been manipulated as follows: flankers orientation (right or left; irrelevant) could be either congruent with the side of the target answer key (for example, a target requiring to press the right side answer key was presented with flankers pointing rightward) or incongruent with the side of the associated answer key (for example, a target requiring to press the right side answer key was presented with flankers pointing leftward). Figure 1b displays an example of experimental stimuli according to the instructions showed in Fig. 1a. The reversed instructions mirrored the schema presented in Fig. 1b and, according to the position of the target answer key, the previous 16 stimuli were replicated, thus obtaining a total of 32 stimuli.

Participants sat approximately at a distance of 70 cm from the computer, were tested in a dimly lit room and viewed the stimuli on a monitor placed with the aid of a chin rest. They began the experimental session reading written instructions and afterwards performed a practice block containing 16 trials. Each trial began with the presentation of a central fixation point (i.e., “•”) for 1000 ms and following by Inter Trial Interval (ITI) for 500 ms. Stimuli appeared immediately after the ITI and remained on screen until subject response. Subsequently, they were simultaneously removed (see Fig. 1c). Subjects were instructed to respond as fast as they could while minimizing their mistakes. Participants completed a first block consisting of 288 trials (16 trials repeated 18 times).

Subsequently participants received written instructions about inverted answer keys and performed a new practice block containing 16 trials and completed another block of 288 trials (16 trials repeated 18 times). All stimuli and conditions were randomized with equal probability. Also, block order was counterbalanced across participants and results between the two blocks were compared as a first control analysis (see results). RTs and correct answers were recorded. Speed, accuracy of response and need to attend to the fixation point were emphasized.

### EEG recordings and data analysis

EEG recordings were performed by means of a net with 128 electrodes (Electrical Geodesics, Inc., version 1.1). Skin/electrode impedance was measured before each EEG recording and kept below 50 kΩ. EEG data were sampled at 500 Hz and processed off-line.

A semiautomatic Independent Component Analysis-based procedure^48^ was applied to identify and to remove cardiac and/or ocular artifacts, as well as activity coming from contraction of head muscles during movement. Data were filtered between 0.1 and 30 Hz and were segmented into epochs of 200 ms before to 500 ms after stimulus presentation. Saturated or corrupted EEG epochs were rejected by visual inspection. EEG channels were re-referenced against the linked mastoids. A 100 ms period in the pre-trigger interval (from −100 to 0 ms) was considered for baseline correction. In order to obtain ERP, about 70 artefact-free epochs corresponding to correct-trial were averaged for each possible triplet of congruent and incongruent conflict. In this way, ERP corresponding to 8 different conditions were obtained: 1) congruent _r_S-R, congruent _A_S-S, congruent _iA_S-R; 2) congruent _r_S-R, congruent _A_S-S, incongruent _iA_S-R; 3) congruent _r_S-R, incongruent _A_S-S, congruent _iA_S-R; 4) congruent _r_S-R, incongruent _A_S-S, incongruent _iA_S-R; 5) incongruent _r_S-R, congruent _A_S-S, congruent _iA_S-R; 6) incongruent _r_S-R, congruent _A_S-S, incongruent _iA_S-R; 7) incongruent _r_S-R, incongruent _A_S-S, congruent _iA_S-R; 8) incongruent _r_S-R, incongruent _A_S-S, incongruent _iA_S-R. Electrode sites for analysis were chosen on the basis of visual inspection of the scalp distributions of ERPs. Previous research demonstrated a N2 component, focal over the fronto-medial locations^13^ and a P3 over central-parietal sites^13^. Inspection of the single-subject ERP and grand averaged waveforms and their topographies confirmed the presence of a fronto-central negativity with a latency in the 200–350 ms interval and a positive component in the 300–450 ms window after stimulus onset (Fig. 3a). Therefore, mean values of the ERP amplitudes were extracted and averaged across the 10 electrodes closest to FCz in the time interval 200–350 ms and across the 10 electrodes closest to Pz in the time interval of the 300–450 ms (Fig. 3b). The latency of the negative peak within the first window was considered as latency of N2 and the N2 amplitude was estimated as difference between the amplitude of the negative peak and the immediately preceding positive peak; the latency of the positive peak within the second window was considered as latency of P3 and the P3 amplitude was estimated as amplitude of the positive peak with respect to the baseline^45,46^.

To assess theta band power modulation under different competing information processing, the time–frequency representation (TFR) was computed for each EEG channel by means of a continuous Complex Morlet transformation^49^ in the range 4–7 Hz, at 1 Hz of frequency resolution. TFR was obtained as the squared magnitude of the complex wavelet-transformed data. The magnitude of complex wavelet transformed EEG channels were averaged in epochs of 1 s from stimulus presentation, with 500 ms of pre-trigger period. Theta band power modulation was quantified as percentage change with respect to the baseline period (Event Related Desyncronization/Synchronization, ERD/ERS^50^):

$${\rm{ERD}}/{\rm{ERS}}={\rm{100}}\,({\rm{Pt}}-{\rm{Pb}})/\mathrm{Pb},$$

where Pt was the TFR value at any given time-frequency values, and Pb the mean power in the baseline period. TFRs were obtain for each of the 8 conditions above defined. Visual inspection of the topography of individual and grand-average of theta band modulation of our data showed a clear theta synchronization in fronto-central sites (Fig. 3c) in the time window of 200–600 ms after stimulus presentation, the average of values of ERS in theta band in the time window of 200–500 ms over the 5 channels closest to FCz were chosen for the statistical analysis.

### EEG and behavioral statistical analysis

A Repeated Measures Analysis of Variance (ANOVA) was performed with a 2 × 2 × 2 statistical design, with the following within factors: _r_S-R (congruent and incongruent), _A_S-S (congruent and incongruent) and _iA_S-R (congruent and incongruent). RTs, accuracies, ERP latencies and amplitudes and Theta power modulation were used as dependent variables in behavioral, ERP and Time-frequency analysis respectively. All statistical analysis were performed by means of the STATISTICA 7 software.

Bonferroni tests were used for post-hoc analyses. Observed power, partial eta squared and confidence intervals for all statistical results were calculated using power calculator included in the STATISTICA software (version 7.0) setting the alpha value at 0.05. Since our goal was to examine mechanisms underlying successful conflict processing, only correct trials have been analyzed. Individual outliers were defined as RTs that deviated more than three SDs from the individual mean latency time and were also removed. Outliers accounted for 4% of the data.

## Data Availability

The datasets generated and analyzed during the current study are available from the corresponding author on reasonable request.
